# Therapeutic hypothermia for encephalopathic newborns with congenital heart defect: A cross-sectional survey on current practices and opinions in Germany

**DOI:** 10.3389/fped.2022.1004086

**Published:** 2022-10-05

**Authors:** Vinzenz Boos, Felix Berger

**Affiliations:** ^1^Department of Congenital Heart Disease, Pediatric Cardiology, German Heart Center Berlin, Berlin, Germany; ^2^Newborn Research, Department of Neonatology, University Hospital Zurich, University of Zurich, Zurich, Switzerland; ^3^German Center for Cardiovascular Research, Congenital Heart Diseases, Berlin, Germany

**Keywords:** congenital heart defect, critical care, neonatal encephalopathy, perinatal asphyxia, therapeutic hypothermia, survey

## Abstract

**Background:**

Therapeutic hypothermia (TH) reduces neonatal mortality and long-term neurodevelopmental impairment in infants with moderate-to-severe hypoxic-ischemic encephalopathy (HIE) caused by perinatal asphyxia. There is an increasing trend to apply TH in other indications and populations, such as infants with mild HIE or neonates with congenital heart defects (CHD), even though there is little evidence to support or refute this.

**Objective:**

The aim of this survey was to analyze practice variations with respect to TH use in neonates with CHD and to assess expert opinions on this topic across tertiary neonatal departments in Germany.

**Methods/Design:**

A web-based survey was sent to all tertiary neonatal departments in Germany. The questionnaire contained 32 multiple-choice questions. The survey inquired current practices on TH in newborns with CHD and expert opinions on various clinical scenarios.

**Main results:**

A total 80 (51.3%) neonatal departments partially completed the survey, and 69 (44.2%) respondents filled out the whole questionnaire. All 80 (100.0%) departments perform TH. TH is offered by 76 (95.0%) respondents to encephalopathic newborns with simple CHD. In infants with critical/complex CHD, TH is offered after perinatal asphyxial HIE and in newborns with encephalopathy after severe acidosis associated with cardiac complications by 25 (31.3%), or 17 (22.1%) respondents, respectively, whereas a clear majority of centers reject TH in these infants. Unclear effects of TH on any ongoing prostaglandin therapy (57.6 and 52.3%, respectively), an increased risk for adverse reactions during TH (51.6 and 52.3%, respectively) and lack of evidence (33.3 and 53.8%, respectively) are the most frequently cited reasons for not performing TH in these infants. The majority of experts from neonatal departments providing comprehensive care for neonates with severe CHD support the initiation of TH in encephalopathic neonates.

**Discussion:**

The considerable heterogeneity in the use of TH in neonates with CHD emphasizes the need for further research to optimize treatment strategies for these patients.

## Introduction

Perinatal asphyxia, around the time of birth, can lead to hypoxic-ischemic encephalopathy (HIE), a common cause of neonatal encephalopathy (NE) associated with high mortality and morbidity among newborns (1–3). Therapeutic hypothermia (TH), induced within 6 h after birth for a duration of 48–72 h improves neurodevelopmental outcomes and has become a key treatment modality for newborns with moderate-to-severe HIE (2). Extended indication for TH in neonates, such as late prematurity, postnatal cardiac arrest, mild HIE, and perinatal arterial ischemic stroke have been proposed, but there is no clear evidence of neurodevelopmental benefit to date (4–7). In addition, the benefit of TH in cases of subacute and chronic ischemia is uncertain (8).

Most randomized controlled trials comparing TH vs. normothermia in NE have excluded newborns with major congenital malformations, including congenital heart defects (CHD) (9). The official guideline of the German-based Society for Neonatology and Pediatric Intensive Care Medicine (GNPI) recommends rejection of TH for perinatal asphyxial HIE in newborns with severe congenital malformations (10). Although it is unclear whether the risk for perinatal asphyxial HIE is increased in newborns with CHD, these infants may equally experience per partum HIE (11, 12). Thus, neonates with CHD and asphyxial HIE may also benefit from TH.

In addition to per partum asphyxial events, neonates with CHD are at increased risk for postnatal events that may lead to NE. Newborns with CHD are at risk for an impaired transition from fetal to extrauterine life. Constriction of the ductus arteriosus in ductal-dependent CHD, insufficient interatrial communication in neonates with dextro-transposition of the great arteries or hypoplastic left heart syndrome, and critical types of obstructive lesions may lead to hemodynamic and oxygenation disorders during the first days of life and potentially contribute to postnatal acidosis. Newborns with CHD more often require postnatal cardiopulmonary resuscitation, including chest compressions (12–15).

Neurodevelopmental deficits or delays are common in infants with severe CHD, and neuroprotective therapies are required to improve neurologic outcome in these high-risk patients (6, 16–18). In addition to acidosis, other mechanisms such as genetics, inflammation, ischemic injuries, perioperative stroke, neurotoxic effects of medications may contribute to cerebral damage in newborns with CHD (19–22). With respect to per partum HIE or NE following postnatal deterioration with severe acidosis, few reports describe the feasibility of TH in neonates with CHD, and there is no evidence to date of long-term benefit of TH in CHD patients (12, 23). However, it is conceivable that despite the lack of sufficient evidence in the literature, neonates with CHD may receive TH for rational considerations.

The objective of this study was to identify practice variations regarding the current use of TH in newborns with CHD in Germany and to assess expert opinions on TH in these high-risk patients.

## Materials and methods

An anonymous online survey was developed to assess the current practice as well as expert opinions on TH in newborns with CHD in tertiary neonatal departments in Germany. The final version of the survey consisted of 34 questions (Supplementary material 1) and was hosted on the web platform SurveyMonkey (SurveyMonkey, San Mateo, USA). No financial incentive was offered for participation in the survey. Institutional Review Board approval was not deemed necessary at each individual institution to which the surveys were sent.

German tertiary neonatal departments were identified *via* www.perinatalzentren.org, a website on perinatal centers in Germany implemented by the Institute for Quality Assurance and Transparency in Health Care (IQTIQ) on behalf of the Federal Joint Committee (G-BA), the highest decision-making body of the joint self-government of physicians, dentists, hospitals and health insurance funds in Germany. The survey was administered *via* email to all 156 clinical directors of level I neonatal units (equivalent to tertiary units, based on the German G-BA nomenclature) in March 2022. Thirty-nine (25%) of the recipients work in facilities belonging to university hospitals. A reminder email was sent in April 2022. The email invitation contained a link to the online questionnaire (in German). The survey was closed in May 2022.

The survey inquired about (A) institutional data, including unit size, availability of pediatric cardiologist services, and use of TH in general; (B) questions on the current use of TH in neonates with simple or severe CHD, or post-cardiac arrest; and (C) personal expert opinions on various clinical scenarios related to newborns with HIE/NE and CHD, as well as personal data on the respondent's clinical experience. Respondents had the choice of remaining anonymous or providing the name of their instutition. Survey questions were pre-dominantly closed-ended, and response options varied, including multiple-choice, single-choice and yes/no answers. Some questions were only available based on specific answers to previous questions. The survey was voluntary and anonymous, with the option to add a comment.

Regarding the cardiac malformations, a classification into simple (such as isolated septal defects or noncritical valve anomalies) and severe (critical and/or complex malformations) CHD was undertaken. The broader term NE encompasses neurological disorders of various causes characterized by an altered level of consciousness, seizures, pathologic tone or reflexes, inability to initiate or maintain respiration, and multi organ dysfunction (3). In the context of perinatal asphyxia, this study uses the historical term HIE, although the term NE better describes the pathological condition at issue in this study. TH following cardiopulmonary resuscitation that occured beyond a successful postnatal transition is referred to as ≪post-resuscitation TH≫. Development of severe acidosis/hypoxia and NE associated with a postnatal cardiac complication (such as heart failure, ductal obstruction, inadequate atrial shunt) related to a heart defect is defined as ≪cardiogenic NE≫. Definition of ≪moderate-to-severe HIE≫ is given in the current German guideline (10).

No questionnaire was excluded from the analysis because of missing data, no answer was excluded. Any missing answers were reported. Responses were exported from www.surveymonkey.com including raw data and summarized data. Analyses were conducted using R studio v1.4.1106 (RStudio, Boston, MA) with R v4.0.4 (The R Foundation for Statistical Computing, Vienna, Austria). Data analysis is largely descriptive. Categorical variables are presented in absolute numbers and percentages. Fisher's exact test was used for comparison of dichotomous variables on practice differences among subgroups in this study. A *p* value < 0.05 was considered statistically significant.

## Results

One hundred and fifty-six survey invitations were delivered to medical directors of all tertiary neonatal departments in Germany. Responses on part A of the survey were received from 80 (51.3%) neonatology departments, and 69 respondents (44.2%) completed all survey questions. Nineteen out of 61 (31.1%) respondents who provided the name of their institution belonged to university hospitals. Institutional characteristics are presented in Table 1. Twenty-seven (33.8%) neonatal units have a dedicated on-site department of pediatric cardiology, but only 12 (15.0%) provide comprehensive treatment for newborns with severe CHD. The majority (76.3%) of units provide no or only primary care until transfer to another center for these patients. All (100%) neonatal units apply TH to neonates with perinatal asphyxial HIE.

**Table 1:** Institutional characteristics of survey respondents.

**Variable**	***n* (%)**
**Number of deliveries**	
< 1000/year	3 (3.8)
1000–1999/year	32 (40.0)
2000–2999/year	29 (36.3)
≥3000/year	16 (20.0)
**Number of neonatal intensive care beds**	
1–10	25 (31.3)
11–20	52 (65.0)
>20	3 (3.8)
**Treatment of newborns with severe congenital heart defect**	
Yes	12 (15.0)
Initial care only, until transfer to a different center	61 (76.3)
No	7 (8.8)
**Number of newborns with severe congenital heart defect**	
None	7 (8.8)
1–10/year	46 (57.5)
11–25/year	20 (25.0)
>25/year	6 (7.5)
I do not know	1 (1.3)
**Availability of pediatric cardiology service**	
No	2 (2.5)
Yes, a pediatric cardiologist is available if needed	51 (63.8)
Yes, on-site department of pediatric cardiology	27 (33.8)
**Therapeutic hypothermia for infants with hypoxic-ischemic encephalopathy**	
Yes	80 (100.0)
No	0 (0.0)
**Number of patients who received therapeutic hypothermia**	
1–2/year	6 (7.5)
3–5/year	34 (42.5)
6–10/year	34 (42.5)
>10/year	4 (5.0)
I do not know	2 (2.5)

Number of respondents: 80.

Neonates with perinatal asphyxia and development of moderate-to-severe HIE would receive TH in 76 (95.0%) centers if simple CHD was present or in 25 (31.3%) centers if severe CHD was present. However, 30 (37.5%), or 19 (23.8%) of these centers had not yet had patients with simple or severe CHD and asphyxial HIE, respectively. No center reported using TH in more than five newborns with simple or severe CHD in the past 5 years. Twelve of 25 (48.0%) centers performing TH in infants with severe CHD have a dedicated pediatric cardiology department, whereas this is the case in only 15 of 55 (27.3%) centers not performing TH (*p* = 0.08).

The majority of 60 (77.9%) units provide TH for neonates without CHD after resuscitation unrelated to the postnatal cardiorespiratory adaptation. Seventeen (22.1%) centers would induce TH in neonates with severe CHD who develop cardiogenic NE. Seven (9.1%) centers have already treated such newborns with TH, whereas the remainder would offer the therapy but have had no such patients in the past 5 years. Twenty-three (31.1%) of the 74 respondents expected a higher risk for adverse events during TH in newborns with severe CHD compared to neonates without CHD, whereas 9 respondents (12.2%) expect a comparable risk and 4 (5.4%) a lower risk. Thirty-eight (51.4%) respondents indicated that they were unable to estimate the risk for adverse events in these cohorts based on their experience (Table 2).

**Table 2:** Therapeutic hypothermia (TH) in infants with congenital heart defect (CHD)—current practice.

**TH practice patterns and indications**	***n* (%)**
**TH for newborns with asphyxial HIE and simple CHD** ^**a**^	
Yes, in the past 5 years:	76 (95.0)
TH would have been induced, but there were no such	30 (37.5)
patients in unit	
1–5 patients	37 (46.3)
6–10 patients	0 (0.0)
>10 patients	0 (0.0)
I do not know the number of patients	9 (11.4)
No	4 (5.0)
**TH for newborns with asphyxial HIE and severe CHD** ^**a**^	
Yes, in the past 5 years:	25 (31.3)
TH would have been induced, but there were no such	19 (23.8)
patients in unit	
1–5 patients	5 (6.3)
6–10 patients	0 (0.0)
>10 patients	0 (0.0)
I do not know the number of patients	1 (1.3)
No	55 (68.8)
**Post-resuscitation TH for newborns without CHD (post delivery-room)** ^**b**^	
Yes	60 (77.9)
No	17 (22.1)
**TH for newborns with cardiogenic NE and severe CHD**^**b**^l	
Yes, in the past 5 years:	17 (22.1)
TH would have been induced, but there were no such	9 (11.7)
patients in unit	
1–5 patients	6 (7.8)
6–10 patients	0 (0.0)
>10 patients	0 (0.0)
I do not know the number of patients	1 (1.3)
No answer	1 (1.3)
No	60 (77.9)
Age at initiation of TH for newborns with cardiogenic NE and severe CHD^*^, **^c^**	
In the delivery room, soon after cardiopulmonary	24 (32.4)
transition	
Within the first 6 h of life	61 (82.4)
Within the first 24 h of life	12 (16.2)
At any time before discharge or transfer	13 (17.6)

HIE, hypoxic-ischemic encephalopathy; NE, neonatal encephalopathy.

a Number of respondents: 80.

b Number of respondents: 77.

c Number of respondents: 74.

* More than one response accepted.

Part C of the survey asked for personal expert opinions, independent of their department's practices (Tables 3, 4). Initiation of TH in neonates with perinatal asphyxial HIE and simple or severe CHD was endorsed by 68 (98.6%), and 31 (44.9%) of responding physicians, respectively. Post-resuscitation TH in newborns without CHD was advocated by 47 (68.1%) of the respondents. One-third of respondents each favored, opposed or were unsure about TH in the case of cardiogenic NE. A majority of respondents considered an increased risk of neurological injury in newborns with perinatal asphyxial HIE (67.7%) and cardiogenic NE (58.1%) as an argument for inducing TH. At the same time, increased risk of adverse reactions and unclear effects of cooling on prostaglandin therapy that may be ongoing argued against TH in patients with asphyxial HIE (56.1% and 57.6%, respectively) or cardiogenic NE (both 52.3%), according to the respondents. Eighteen of 22 (81.8%) experts from centers performing TH in newborns with asphyxial HIE and severe CHD were in favor of this therapy, whereas only 13 of 47 (27.7%) of experts advocated TH in centers not performing TH in this scenario (*p* = < 0.0001).

**Table 3:** Expert opinions on therapeutic hypothermia (TH) in infants with congenital heart defect (CHD).

**Survey respondents' opinions and clinical experience**	***n* (%)**
In which neonates with perinatal asphyxial HIE would you consider applying TH?*	
Newborns without any congenital malformations	68 (98.6)
Newborns with non-cardiac congenital malformations	57 (82.6)
Newborns with simple CHD (such as isolated septal	69 (100.0)
efects or non-critical valve pathologies)	
Newborns with severe CHD (critical and/or complex	38 (55.1)
lesions)	
None of these infants	0 (0.0)
**Would you initiate TH in a newborn with perinatal asphyxial HIE and simple CHD?**	
Yes	68 (98.6)
Uncertain	1 (1.4)
No	0 (0.0)
**Would you initiate TH in a newborn with perinatal asphyxial HIE and severe CHD?**	
Yes	31 (44.9)
Uncertain	24 (34.8)
No	14 (20.3)
**Would you initiate post-resuscitation TH for newborns without CHD (post delivery-room)?**	
Yes	47 (68.1)
Uncertain	11 (15.9)
No	11 (15.9)
**Would you initiate TH in a newborn with cardiogenic NE and severe CHD?**	
Yes	24 (34.8)
Uncertain	23 (33.3)
No	22 (31.9)
**Opinion on the existing data on TH in encephalopathic neonates with severe CHD?**	
Satisfactory	3 (4.3)
Not satisfactory, but not required	8 (11.6)
Not satisfactory, more studies are needed	58 (84.1)
Specialty/subspecialty of respondent^*^	
Neonatologist	65 (94.2)
Pediatric cardiologist	20 (29.0)
Pediatric intensivist	36 (52.2)
No pediatric subspecialisation	0 (0.0)
Anesthesiologist	2 (2.9)
Workplace of the respondent^*^	
Neonatal intensive care unit (NICU)	28 (40.6)
Mixed neonatal-and-pediatric intensive care unit	45 (65.2)
(NICU/PICU)	
Pediatric intensive care unit (PICU)	10 (14.4)
Others	2 (2.9)
**Neonatal department of a university hospital**	
Yes	22 (31.9)
No	47 (68.1)
**Duration of clinical experience of respondent in neonatal intensive care**	
0–5 years	1 (1.4)
6–10 years	15 (21.7)
11–20 years	19 (27.5)
>20 years	34 (49.3)

Number of respondents: 69.

HIE, hypoxic-ischemic encephalopathy; NE neonatal encephalopathy.

* More than one response accepted.

**Table 4:** Expert opinions on reasons for and against therapeutic hypothermia (TH) in infants with congenital heart defect (CHD).

**Survey respondents' arguments for and against TH**	**Scenario 1**	**Scenario 2**	**Scenario 3**
	**Perinatal asphyxial HIE and simple CHD**	**Perinatal asphyxial HIE and severe CHD**	**Cardiogenic NE and severe CHD**
**Which might be the reasons for TH in neonates with…** (*Scenario*1, 2, 3)^*^	***n*** **=** **67**	***n*** **=** **65**	***n*** **=** **62**
Newborns with CHD have an increased risk for neurological injury	13 (19.4)	44 (67.7)	36 (58.1)
Findings on children without CHD can be rationally applied to this scenario	59 (88.1)	30 (46.2)	26 (41.9)
There is sufficient evidence to support TH in this scenario	15 (22.4)	2 (3.1)	2 (3.2)
Adverse reactions can be treated adequately	35 (52.2)	15 (23.1)	13 (21.0)
**Which reasons might argue against TH in neonates with…** (*Scenario*1, 2, 3)^*^	***n*** **=** **57**	***n*** **=** **66**	***n*** **=** **65**
Newborns with CHD have no increased risk of neurological injury	6 (10.5)	2 (3.0)	1 (1.5)
Findings on children without CHD should not be applied to this scenario	9 (15.8)	18 (27.3)	18 (27.7)
There is no evidence to support TH in newborns in this scenario	17 (29.8)	22 (33.3)	35 (53.8)
Newborns in this scenario are at increased risk for adverse reactions during TH	14 (24.6)	37 (56.1)	34 (52.3)
The effects of TH on any ongoing prostaglandin therapy are unclear	N/A	38 (57.6)	34 (52.3)

HIE, hypoxic-ischemic encephalopathy; N/A, not available; NE, neonatal encephalopathy.

* More than one response accepted.

Most respondents of part C were neonatologists (94.2%), pediatric intensivists (52.2%), and/or pediatric cardiologists (29.0%). Two-thirds (65.2%) of respondents worked in a combined neonatal and pediatric intensive care unit, and/or 40.6% in a dedicated neonatal intensive care unit. Their clinical experience in neonatal intensive care was distributed among four age groups (number of years one has been working in neonatal intensive care) as follows: 1.4% (0–5 years), 21.7% (6–10 years), 27.5% (11–20 years), 49.3 % (>20 years).

Neonatal departments providing comprehensive care for neonates with severe CHD are more likely to initiate TH in newborns with asphyxial HIE and severe CHD (66.7 vs. 25.0%, *p* = 0.0071) and in infants with cardiogenic NE (70.0 vs. 14.9%, *p* = 0.0006) compared to centers providing only primary or no care for these infants. In addition, the majority of experts from these centers favor the induction of TH after asphyxial HIE in neonates with simple or severe CHD and in infants with cardiogenic NE (Table 5).

**Table 5:** Differences between neonatal units with and without cardiologic care for newborns with severe congenital heart defect (CHD).

	**Pediatric cardiologic care for severe CHD**	**Not or only primary care for severe CHD**	***p*-value**
**Institutional approach**	***n*** **=** **12**	***n*** **=** **68**	
TH for newborns with asphyxial HIE and simple CHD	12 (100.0%)	64 (94.1%)	1,0000
TH for newborns with asphyxial HIE and severe CHD	8 (66.7%)	17 (25.0%)	0,0071
Post-resuscitation TH for newborns without CHD (post delivery-room)^a^	8 (80.0%)	52 (77.6%)	1,0000
TH for newborns with cardiogenic NE and severe CHD^a^	7 (70.0%)	10 (14.9%)	0,0006
**Personal expert opinion**	***n*** **=** **9**	***n*** **=** **60**	
Would initiate TH in a newborn with perinatal asphyxial HIE and simple CHD	8 (88.9%)	60 (100.0)	0,1304
Would initiate TH in a newborn with perinatal asphyxial HIE and severe CHD	6 (66.7%)	25 (41.7%)	0,2810
Would initiate post-resuscitation TH for newborns without CHD (post delivery-room)	6 (66.7%)	41 (68.3%)	1,0000
Would initiate TH in a newborn with cardiogenic NE and severe CHD	5 (55.6%)	19 (31.7%)	0,2590

HIE, hypoxic-ischemic encephalopathy; NE, neonatal encephalopathy; TH, therapeutic hypothermia.

a Number of respondents: 10, and 67, respectively.

## Discussion

This is the first survey to report on perceived practices and opinions about TH in neonates with CHD in tertiary neonatal units. The results demonstrate considerable variability among clinicians regarding TH in encephalopathic neonates with severe CHD. A simple CHD had almost no influence on the application of TH in neonates with asphyxial HIE. In the presence of a severe cardiac malformation, initiation of TH was significantly less frequent, both in per partum HIE and in the case of a postnatal event. Fear of adverse effects and lack of evidence were the most commonly cited reasons for not applying TH in these infants.

A large majority of 95.0% favored the initiation of TH in neonates with perinatal asphyxia and development of moderate-to-severe HIE who had simple CHD. Although TH has not been adequately studied in infants with simple CHD, 88.1% of respondents argued that the results in infants without CHD can be rationally applied to infants with simple CHD. In fact, 22.4% of respondents stated that there was sufficient evidence to support the use of TH in these patients. In newborns with simple cardiac malformations such as septal defects or noncritical valve pathologies, the opinion of respondents is almost unanimous in favor of performing TH despite the lack of evidence.

In contrast, practices and opinions vary widely regarding infants with severe CHD. Although only few cases of neonates with severe CHD treated with TH have been published, and infants with (major) congenital anomalies have been excluded in many randomized trials on TH, even neonates with severe CHD are offered TH in a variety of clinical scenarios in some centers (9, 12, 23). Almost one-third of departments offer TH after perinatal asphyxia in neonates with severe CHD, but when asked for their personal opinion, only 20.3% of the respondents opposed TH. Surprisingly, even a quarter of all neonatology departments that do not provide treatment for neonates with severe CHD at all or only until transfer to another center offer TH in these patients. In the case of postnatal deterioration due to a cardiac complication resulting from the heart defect, the answers were similar. While 22.1% of centers offer TH after cardiogenic NE, as many as one-third of respondents would be in favor of performing TH in this situation or undecided, respectively. There is a remarkable discrepancy between institutional experience and personal opinion. It is unclear why the majority of respondents from centers with comprehensive treatment of newborns with severe CHD support TH in infants with HIE and cardiogenic. We can only speculate whether more experience in treating neonates with CHD, a high level of awareness of potential neurodevelopmental impairments in these infants, or the belief that adverse effects can be adequately treated play a role in this.

The majority of experts state that newborns with severe CHD are at increased risk for adverse effects during TH. Perinatal asphyxia is associated with injury to the heart, including myocardial ischemia, ventricular dysfunction, arrhythmias, hypotension, and persistent pulmonary hypertension of the newborn (24). Hypoxia and acidosis due to poor cardiac perfusion further exacerbates pulmonary hypertension by increasing pulmonary vascular resistance (25). In addition, TH negatively affects cardiovascular dysfunction by leading to peripheral vasoconstriction, reduction in cardiac output, increase in pulmonary vascular resistance, prolongation of the QTc interval, and bradycardia (25, 26). Although TH primarily targets neuroprotection, the myocardial damage may decrease with TH, suggesting a cardioprotective effect in term asphyxiated neonates (27). Not surprisingly, awareness of potential adverse effects of TH on neonates with CHD, who are already more likely to have disturbed hemodynamics, is high because of to the multiple influences of acidosis, asphyxia and hypoxia, as well as TH, on the cardiovascular system (13).

The lack of evidence for TH in neonates with HIE outside of standard inclusion and exclusion criteria leads to large differences between individual centers in the treatment options offered. Burnsed et al., in a 2016 case-based survey among practicing neonatologists in the United States, reported significant differences in opinion and that the use of TH is widespread in patients who have not been studied in randomized controlled trials. Thirty-nine percent of respondents would offer TH to late preterm infants with asphyxial HIE, and 36% would offer TH to a newborn after cardiac arrest at 24 h of age (7). A 2018 survey of cooling centers in the United Kingdam found that a large number of centers offer TH to neonates with only mild HIE (28). It appears that TH is increasingly being used in neonates with indications other than those on which randomized trials of TH are based (2, 9). This may be due to the fact that TH is the only therapeutic intervention for NE with beneficial neurologic outcomes to date, although this effect has been proven only in neonates with NE secondary to an intrapartum asphyxia event (2, 29). Recent studies indicate that TH may have a neuroprotective effect even in cases of only mild encephalopathy (4, 30).

The official German guideline for the treatment of neonatal asphyxia recommends excluding children with severe congenital malformations including CHD from TH (10). However, the results of this survey show that TH is offered in neonates with severe CHD in numerous centers and that only a minority of experts oppose TH after asphyxial HIE or cardiogenic NE in these infants. The aforementioned guideline was published in 2013 and is currently being revised (10, 31). It remains to be seen whether severe congenital malformations in general will again be listed as a strict reason for exclusion, considering that in practice newborns with these malformations are already offered TH in some centers.

While the criteria for perinatal asphyxia and HIE are clearly defined in the guideline, clear definitions regarding timing and diagnosis are lacking in the case of post-resuscitation TH or cardiogenic NE (10). The lack of clear diagnostic criteria and indications in these cases may be responsible for the fact that TH is not offered to infants with postnatal events in many centers. The current trend toward targeted temperature management to maintain normothermia and avoid fever rather than initiation and maintenance of hypothermia in resuscitated adults may also influence neonatologists' attitudes toward TH in post partum events (32).

The present study has some limitations. It is difficult to completely prevent or estimate selection and response bias. Although responses were collected from about half of the invited neonatal units, this study does not represent the practices and opinions of all tertiary neonatal departments in Germany. We cannot exclude differences regarding TH experience, treatment of CHD patients or differences in diagnosis of HIE/NE between responder centers to non-responder centers. Some respondents only partially answered the survey. The selection of survey participants from neonatal departments means that the experiences and opinions of physicians from departments of pediatric cardiology or pediatric intensive care, who may also treat these patients in the neonatal period, could not be captured. However, the restriction of the survey to neonatal units can be justified by the extensive experience of these facilities with TH in newborns.

In conclusion, there was broad consensus that simple CHD should not influence the use of TH in the neonatal period. In contrast, treatment practices for TH in neonates with severe CHD varied considerably among tertiary neonatal departments in Germany. Further research is required to investigate the causes for preoperative encephalopathy in newborns with CHD and to assess whether TH can improve neurodevelopmental outcomes in these patients. Given the rarity of these events, prospective, randomized, multicenter trials should be preferred.

## Data availability statement

The raw data supporting the conclusions of this article will be made available by the authors, without undue reservation.

## Author contributions

VB designed the questionnaire, conducted the survey, collected, analyzed and interpreted data, and wrote the first draft of the manuscript. FB contributed to conceptualization, data interpretation, and critically revised the manuscript. Both authors contributed to the article and approved the submitted version.

## Funding

VB financed the online survey.

## Conflict of interest

The authors declare that the research was conducted in the absence of any commercial or financial relationships that could be construed as a potential conflict of interest.

## Publisher's note

All claims expressed in this article are solely those of the authors and do not necessarily represent those of their affiliated organizations, or those of the publisher, the editors and the reviewers. Any product that may be evaluated in this article, or claim that may be made by its manufacturer, is not guaranteed or endorsed by the publisher.
