# Supplementary material for: Therapeutic hypothermia for encephalopathic newborns with congenital heart defect: A cross-sectional survey on current practices and opinions in Germany
Source: Front Pediatr. 2022 Oct 5;10:1004086. doi: 10.3389/fped.2022.1004086 (PMC9581247; doi:10.3389/fped.2022.1004086)
Supplement: Supplementary file 1 [file Data_Sheet_1.PDF]

Supplementary Material 1

Survey Questions

A1. How many newborns are born in your hospital per year?
a) <1000 / year
b) 1000-1999 / year
c) 2000-2999 / year
d) ≥3000 / year
e) I do not know

A2. What is the number of neonatal intensive care beds in your unit?
a) 1-10
b) 11-20
c) >20
d) I do not know

A3. Does your department provide care for newborns with severe forms of congenital heart defects (critical and/or complex malformations)?
a) Yes
b) Yes, only primary care, until transfer to another center
c) No

A4. How many newborns with severe forms of congenital heart defects (critical and/or complex malformations) are treated in your department per year?
a) None
b) 1-10 / year
c) 11-25 / year
d) >25 / year
e) I do not know

A5. Does your department have a pediatric cardiology service for emergency consultations for children with congenital heart defects?
a) No
b) Yes, a pediatric cardiologist is available if needed
c) Yes, a pediatric cardiology department is part of the center where the unit is located

A6. Does your department provide therapeutic hypothermia for neonates with perinatal asphyxia and moderate to severe hypoxic-ischemic encephalopathy (HIE)?
a) Yes
b) No *→ Continue with question C1*

A7. Approximately how many newborns per year are treated with therapeutic hypothermia?
a) 1-2 / year
b) 3-5 / year
c) 6-10 / year
d) >10 / year
e) I do not know

B1. Does your department provide therapeutic hypothermia for neonates
- with simple congenital heart defects (such as isolated septal defects or noncritical valve anomalies)
AND
- perinatal asphyxia (e.g., due to uterine rupture or placental abruption) with development of moderate to severe hypoxic-ischemic encephalopathy (HIE)?
a) Yes
b) No

B2. *If the previous answer was yes:* Approximately how many newborns with simple congenital heart defects (such as isolated septal defects or noncritical valve anomalies) have been treated with therapeutic hypothermia in your department in the last five years?
a) The therapy would have been initiated, but there were no such patients in our department
b) 1-5
c) 6-10
d) >10
e) I do not know

B3. Does your department provide therapeutic hypothermia for neonates
- with severe congenital heart defects (critical and/or complex malformations) AND
- perinatal asphyxia (e.g., due to uterine rupture or placental abruption) with development of moderate to severe hypoxic-ischemic encephalopathy (HIE)?
a) Yes
b) No

B4. *If the previous answer was yes:* Approximately how many newborns with severe congenital heart defects (critical and/or complex malformations) have been treated with therapeutic hypothermia in your department in the last five years?
a) The therapy would have been initiated, but there were no such patients in our department
b) 1-5
c) 6-10
d) >10
e) I do not know

B5. Does your department provide therapeutic hypothermia for neonates
- without congenital heart defects AND
- without perinatal asphyxia (no fetal acidosis, no birth-related complications) AND
- with development of severe acidosis and moderate to severe hypoxic-ischemic encephalopathy (HIE) after resuscitation unrelated to postnatal cardiorespiratory adaptation?
a) Yes
b) No

B6. Does your department provide therapeutic hypothermia for neonates
- with severe congenital heart defects (critical and/or complex malformations) AND
- without perinatal asphyxia (no fetal acidosis, no birth-related complications) AND
- with development of severe acidosis and moderate to severe hypoxic-ischemic encephalopathy (HIE) as part of a postnatal cardiac complication (such as heart failure, ductal obstruction, inadequate atrial shunt), which is related to the heart defect?
a) Yes
b) No

B7. *If the previous answer was yes:* Approximately how many newborns with severe congenital heart defects (critical and/or complex malformations) who develop acidosis and HIE as part of a postnatal cardiac complication which is related to the heart defect have been treated with therapeutic hypothermia in your department in the last five years?
a) The therapy would have been initiated, but there were no such patients in our department
b) 1-5
c) 6-10
d) >10
e) I do not know

B8. At what age would the diagnosis of acidosis and HIE (associated with a postnatal cardiac complication) justify the initiation of therapeutic hypothermia in neonates with severe congenital heart defects (critical and/or complex malformations)? (Check all that apply)
a) In the delivery room, shortly after cardiorespiratory adaptation
b) Within the first 6 hours of life
c) Within the first 24 hours of life
d) At any time prior to discharge or transfer
e) Other, please specify:

B9. In your clinical experience, what is the risk for relevant adverse events during therapeutic hypothermia in neonates with severe congenital heart defects (critical and/or complex malformations), as compared to neonates without a congenital heart defect?
a) Higher
b) Comparable
c) Lower
d) I cannot compare the risk for relevant adverse events in these cohorts based on my clinical experience

C: Regardless of the practice in your department, please give your personal expert opinion on the following questions

C1. In which neonates with perinatal asphyxia and signs of moderate to severe HIE would you personally consider applying therapeutic hypothermia? (Check all that apply)
a) Newborns without any congenital malformations
b) Newborns with noncardiac congenital malformations
c) Newborns with simple congenital heart defects (such as isolated septal defects or noncritical valve anomalies)
d) Newborns with severe congenital heart defects (critical and/or complex malformations)
e) None of these newborns
f) Other, please specify:

C2. Would you initiate therapeutic hypothermia in a neonate
- with a simple congenital heart defect (such as isolated septal defects or noncritical valve anomalies) AND
- perinatal asphyxia (e.g., due to uterine rupture or placental abruption) with development of moderate to severe hypoxic-ischemic encephalopathy (HIE)?
a) Yes
b) Uncertain
c) No

C3. What might be the reasons for therapeutic hypothermia in neonates with simple congenital heart defects (such as isolated septal defects or noncritical valve anomalies)? (Check all that apply)
a) Newborns with congenital heart defects are at increased risk for neurological injury
b) Findings on children without heart defects can be rationally applied to children with simple congenital heart defects
c) There is sufficient evidence to support hypothermia in neonates with simple congenital heart defects
d) Adverse reactions can be treated adequately
e) Other, please specify:

C4. What reasons might argue against therapeutic hypothermia in neonates with simple congenital heart defects (such as isolated septal defects or noncritical valve anomalies)? (Check all that apply)
a) Newborns with congenital heart defects are not at increased risk for neurologic injury
b) Findings on children without heart defects should not be applied to children with simple heart defects
c) There is no evidence to support hypothermia in neonates with a simple congenital heart defect
d) Neonates with a simple congenital heart defect are at increased risk for adverse reactions during hypothermia
e) Other, please specify:

C5. Would you initiate therapeutic hypothermia in a neonate
- with a severe congenital heart defect (critical and/or complex malformations) AND
- perinatal asphyxia (e.g., due to uterine rupture or placental abruption) with development of moderate to severe hypoxic-ischemic encephalopathy (HIE)?
a) Yes
b) Uncertain
c) No

C6. What might be the reasons for therapeutic hypothermia in neonates with severe congenital heart defects (critical and/or complex malformations)? (Check all that apply)

a) Newborns with congenital heart defects are at increased risk for neurological injury
b) Findings on children without heart defects can be rationally applied to children with severe congenital heart defects
c) There is sufficient evidence to support hypothermia in neonates with severe congenital heart defects
d) Adverse reactions can be treated adequately
e) Other, please specify:

C7. What reasons might argue against therapeutic hypothermia in neonates with severe congenital heart defects (critical and/or complex malformations)? (Check all that apply)
a) Newborns with congenital heart defects are not at increased risk for neurologic injury
b) Findings on children without heart defects should not be applied to children with severe heart defects
c) There is no evidence to support hypothermia in neonates with a severe congenital heart defect
d) Neonates with a severe congenital heart defect are at increased risk for adverse reactions during hypothermia
e) The effects of hypothermia on any ongoing prostaglandin therapy are unclear
f) Other, please specify:

C8. Would you initiate therapeutic hypothermia in a neonate
- without congenital heart defects AND
- without perinatal asphyxia (no fetal acidosis, no birth-related complications) AND
- with development of severe acidosis and moderate to severe hypoxic-ischemic encephalopathy (HIE) after resuscitation unrelated to postnatal cardiorespiratory adaptation?
a) Yes
b) Uncertain
c) No

C9. Would you initiate therapeutic hypothermia in a neonate
- with severe congenital heart defects (critical and/or complex malformations) AND
- without perinatal asphyxia (no fetal acidosis, no birth-related complications) AND
- with development of severe acidosis and moderate to severe hypoxic-ischemic encephalopathy (HIE) in the context of a postnatal cardiac complication (such as heart failure, ductal obstruction, inadequate atrial shunt), which is related to the heart defect?
a) Yes
b) Uncertain
c) No

C10. What might be the reasons for therapeutic hypothermia in neonates with severe congenital heart defects (critical and/or complex malformation) who develop acidosis and HIE as part of a postnatal cardiac complication? (Check all that apply)
a) Newborns with congenital heart defects are at increased risk for neurological injury
b) Findings on children without heart defects can be rationally applied to children with severe congenital heart defects who develop acidosis and HIE due to a cardiac complication
c) There is sufficient evidence to support hypothermia in neonates with severe congenital heart defects who develop acidosis and HIE due to a cardiac complication
d) Adverse reactions can be treated adequately
e) Other, please specify:

C11. What reasons might argue against therapeutic hypothermia in neonates with severe congenital heart defects (critical and/or complex malformation) who develop acidosis and HIE as part of a postnatal cardiac complication? (Check all that apply)
a) Neonates with congenital heart defects and acidosis as part of a cardiac complication are not at increased risk for neurologic damage
b) Findings on children without heart defects should not be applied to children with severe congenital heart defects and development of acidosis as part of a cardiac complication
c) There is no evidence for hypothermia in neonates in this situation
d) Neonates with a severe congenital heart defect and acidosis due to a cardiac complication are at increased risk for adverse reactions during hypothermia
e) The effects of hypothermia on any ongoing prostaglandin therapy are unclear
f) Other, please specify:

C12. What is your opinion of the existing data on therapeutic hypothermia in encephalopathic neonates with severe congenital heart defects (critical and/or complex malformation)?
a) Satisfactory
b) Not satisfactory, but not required
c) Not satisfactory, further studies are needed

C13. What is your clinical specialization / subspecialization? (Check all that apply)
a) Neonatology
b) Pediatric Cardiology
c) Pediatric Intensive Care
d) Pediatric and Adolescent Medicine
e) Anesthesiology
f) Other, please specify:

C14. What type of unit do you work in? (Check all that apply)
a) Neonatal intensive care unit (NICU)
b) Mixed neonatal and pediatric intensive care unit (NICU/PICU)
c) Pediatric intensive care unit (PICU)
d) Other, please specify:

C15. Is your department part of a university hospital?
a) Yes
b) No

C16. What is your clinical experience in neonatal intensive care?
a) 0-5 years
b) 6-10 years
c) 11-20 years
d) >20 years

C17. What is the name of your department? (NOTE: This information will remain anonymous and will only be used to avoid reminder letters or, if necessary, to combine data from multiple respondents at the same department).
(open answer)

C18. Comments on the survey
(open answer)

Thank you for taking the time to participate in the survey.
